# Resveratrol protects against isoflurane-induced testicular injury: a stereological and molecular study in mice

**DOI:** 10.1016/j.clinsp.2026.100878

**Published:** 2026-02-19

**Authors:** Zahra Mohammadi, Sanaz Alaee, Majid Kamali-Dolat Abadi, Somayyeh Karami-Mohajeri, Zahra Khodabandeh, Saeed Shokri, Sulagna Dutta, Pallav Sengupta, Hesam Kamyab

**Affiliations:** a Department of Toxicology and Pharmacology, Faculty of Pharmacy, Kerman University of Medical Sciences, Kerman, Iran; b Department of Reproductive Biology, School of Advanced Medical Sciences and Technologies, Shiraz University of Medical Sciences, Shiraz, Iran; c Stem Cell Technology Research Center, Shiraz University of Medical Sciences, Shiraz, Iran; d Infertility Research Centre, Shiraz University of Medical Sciences, Shiraz, Iran; e Applied Physiology Research Center, Cardiovascular Research Institute, Department of Physiology, School of Medicine, Isfahan University of Medical Sciences, Isfahan, Iran; f Pharmaceutics Research Center, Institute of Neuropharmacology, Kerman University of Medical Sciences, Kerman, Iran; g School of Medical Sciences, Faculty of Medicine and Health, University of Sydney, Sydney, New South Wales, Australia; h Basic Medical Sciences Department, College of Medicine, Ajman University, Ajman, UAE; i Department of Biomedical Sciences, College of Medicine, Gulf Medical University, Ajman, UAE; j Universidad UTE, Quito 170527, Ecuador; k Department of Biomaterials, Saveetha Dental College and Hospital, Saveetha Institute of Medical and Technical Sciences, Chennai 600077, India; l Department of Medical Nanotechnology, School of Advanced Medical Sciences and Technologies, Shiraz University of Medical Sciences, Shiraz, Iran

**Keywords:** Resveratrol, Isoflurane, Gene expression, Stereology, Seminiferous tubule, Oxidative Stress

## Abstract

**Background:**

This study examines the therapeutic effects of resveratrol, known for its antioxidant and anti-inflammatory properties, in mitigating the harmful effects of isoflurane, an inhalation anesthetic, on testicular tissues in mice.

**Methods:**

Sixty adult male C57BL/6 mice were grouped into six categories: control (normal saline), isoflurane only (1.5% for 1-hour/day), low-dose Resveratrol (RSV, 50 mg/kg/day), high-dose RSV (100 mg/kg/day), isoflurane combined with low-dose RSV, and isoflurane combined with high-dose RSV. After 35-days, the mice were euthanized, and their testes were harvested for stereological analysis and the assessment of antioxidant and apoptotic markers.

**Results:**

Exposure to isoflurane resulted in decreased seminiferous tubule volume and length, along with increased interstitial tissue volume of the testis. Co-treatment with resveratrol, especially at higher doses, mitigated these changes. Additionally, resveratrol modulated the expression of the apoptotic genes *Bcl2l1, Bax*, and *Caspase-3*, and enhanced the expression of antioxidant genes *GPX1, Sod1*, and *catalase* in mice that received isoflurane.

**Conclusions:**

The findings suggest that resveratrol effectively counters isoflurane-induced damage in testicular structures and gene expression, enhancing tubular integrity and antioxidant defense. This indicates a potential therapeutic approach to protect against isoflurane-induced testicular toxicity.

## Highlights

• Resveratrol studied for protection against isoflurane-induced testicular damage.
• Sixty C57BL/6 mice were divided into control, isoflurane, and resveratrol groups.
• Isoflurane reduced testicular volume, tubular structures, and altered cells.
• Resveratrol modulated apoptotic markers and boosted antioxidant gene expression.
• Resveratrol shows potential in preventing isoflurane-induced testicular toxicity.

## Introduction

The use of inhalation anesthetics is ubiquitous in anesthesia management. Isoflurane, sold under the brand name Forane and also known as 1‑chloro-2,2,2-trifluoroethyl difluoromethyl ether or isoflurane, is unique among anesthetics because it is administered through inhalation and primarily removed from the body via the respiratory system.^1^ This stable, non-explosive gas is commonly used in operating rooms, recovery areas, and veterinary clinics.^2^ As a halogenated volatile anesthetic, isoflurane is favored in surgical environments for its ability to control the depth of anesthesia, producing amnesia, sedation, and hypnosis in a dose-dependent manner.^1^ In contrast to intravenous anesthetics, inhalation anesthetics like isoflurane act on multiple critical sites within the central nervous system and other body systems.^3^ Occupational exposure to anesthetic gases has been associated with genetic damage. Even minute concentrations of waste anesthetic gases can elevate genetic damage risks.^4^ Isoflurane has been implicated in impairing seminiferous tubules and spermatogenesis due to disruptions in sexual hormone balance.^5^

Isoflurane exposure has been linked to oxidative stress due to its ability to generate Reactive Oxygen Species (ROS).^5^ ROS initiate pathways that contribute to oxidative damage. These ROS can induce damage to lipids, proteins, and DNA, which may result in cell damage and programmed cell death, known as apoptosis.^6^ Therefore, oxidative stress is considered a significant mechanism underlying isoflurane's adverse effects. Therefore, mitigating oxidative stress may hold promise in reducing the toxicity associated with isoflurane exposure.^7^

Male infertility can result from low sperm production, poor sperm motility, abnormal sperm shape, or blockages in the reproductive tract. Hormonal imbalances, genetic factors, infections, lifestyle habits, and environmental toxins also play a role.^8^^,^^9^

Natural antioxidants derived from plants have shown promise in ameliorating the toxic effects of various compounds on the reproductive system and infertility-related conditions.^10^, ^11^, ^12^ Studies have demonstrated that plant-based antioxidants, such as flavonoids, polyphenols, and vitamins, possess potent antioxidant properties, effectively scavenging ROS and reducing oxidative stress in reproductive tissues.^13^, ^14^, ^15^ This is particularly significant as oxidative stress has been implicated in the pathogenesis of reproductive disorders and infertility by damaging sperm, oocytes, and reproductive organs.^16^ It is suggested that harnessing the antioxidant potential of plant compounds may offer therapeutic strategies for mitigating the adverse effects of toxins on the reproductive system and addressing infertility issues.^10^

The phytoalexin polyphenol resveratrol (trans-3,4,5-trihydroxystilbene) is found in plants such as grapes, mulberries, peanuts, and rhubarb. In the body, resveratrol is readily absorbed, rapidly metabolized, and mainly eliminated through urination.^17^ As well as having antioxidant and anti-inflammatory properties, resveratrol is also anticancer, antimicrobial, anti-neurodegenerative, and estrogenic.^18^ It is known that resveratrol has anti-oxidant properties in that it scavenges ROS such as hydroxyl radicals, superoxide radicals, and metal-induced radicals.^19^ The antioxidative properties of resveratrol are attributed to its high redox properties, which help scavenge free radicals. The antioxidant properties of resveratrol are activated by many enzymes, including catalase and superoxide dismutase.^20^ Resveratrol appears to be beneficial to both human and animal reproduction, according to a large amount of research. Research indicates that resveratrol enhances mitochondrial membrane potential, sperm motility, and viability, and protects spermatocytes from lipid peroxidation.^21^ A notable property of resveratrol is its capacity to inhibit the formation of Reactive Oxygen Species (ROS) and protect normal cells from DNA damage and apoptosis by modulating antiapoptotic mediators (*Bcl2l1*) and suppressing proapoptotic mediators (*Bax*, cytochrome C, and caspases 3/9).^22^

According to the established protective properties of resveratrol, this study aims to investigate the adverse effects of isoflurane on male reproductive health by assessing its impact on testicular morphology, specifically interstitial and seminiferous tubule volume and length, cellular composition, and the expression of apoptosis and antioxidant-related genes. A central objective is to evaluate the efficacy of both high and low-dose resveratrol in mitigating this toxicity, thereby elucidating its potential as a therapeutic intervention against isoflurane-induced testicular damage.

## Materials and methods

### Study animals

This research involved 60 C57BL/6 mice, each weighing between 25 g and 30 g, obtained from the laboratory animal center at Shiraz University of Medical Sciences. The mice were kept under controlled humidity and temperature conditions, with unrestricted access to food and water. All experimental procedures involving animals were approved by the Animal Ethics Committee of Shiraz University of Medical Sciences (Approval ID: IR.SUMS.AEC.1402.060) and conducted in compliance with the ARRIVE guidelines (Animal Research: Reporting of In Vivo Experiments) to ensure rigorous reporting, transparency, and reproducibility.

### Treatments

After a two-week acclimatization period, the mice were randomly divided into six experimental groups (*n* = 10). The control group received daily injections of normal saline. The isoflurane group was exposed to 1.5 % isoflurane for one hour daily in an adjustable inhalation chamber. Two groups served as resveratrol controls and received either a low (50 mg/kg/day) or a high (100 mg/kg/day) dose intraperitoneally. The final two groups received combined treatments, being administered either the low or high dose of resveratrol intraperitoneally and one hour of isoflurane exposure. The regimen was administered for five consecutive days per week over a total experimental period of 35 days.

### Stereological study of the testis

Under anesthesia with a CO_2_ chamber, the left testis was removed on the last day of the experiment. To evaluate the tubular and interstitial tissue volume of the testis, the primary volume of the testis was measured using the immersion method,^23^ and the final volume was estimated after tissue staining and processing. Sections were obtained isotropically and uniformly at random by employing the Orientator method. With the tissue oriented, eight to ten slabs were collected. The diameter and area of the testis were determined by punching a circular piece from a slab with a trocar. The cut surfaces of the slabs and circular pieces were embedded in paraffin blocks. Sections of 5 µm and 20 µm thickness were obtained by advancing the microtome. The number density of different types of cells was calculated using 20 µm sections, while 5 µm-thick sections were utilized for volume estimation of specific items. Staining was performed using hematoxylin and eosin. Following staining, the surface area of the circular piece was measured again, and volume shrinkage^24^ was calculated by using the formula: $Volume\;shrinkage\;=\;1-\;(\frac{AA}{AB})1.5$; where AA and AB denote the areas of the circular piece after and before processing, sectioning, and staining, respectively. Volume shrinkage is the proportional decrease in tissue volume caused by the histological processing. The final volume of the testis was estimated using the formula: Vfinal=Vprimary×(1−volumeshrinkage); Vfinal is the corrected total testis volume after accounting for tissue shrinkage. The densities of the structural parameters were estimated, and the total amount of each parameter was obtained by multiplying the density by “V_final_”. Microscopic analysis was conducted using video microscopy, which involved a microscope (E-200, Nikon, Japan) connected to a video camera, a computer, and a flat-screen monitor. In each testis, 10 to 14 microscopic fields were examined to estimate each parameter. Microscopic fields were selected using systematic random sampling. An X- and Y-axis stage micrometer was used to move the slide at equal intervals. The relevant grids (test probes) were overlaid on the monitor by the stereology software developed at the Morphometry and Stereology Research Centre, Shiraz University of Medical Sciences, Shiraz, Iran.

### Measurement of tubular and interstitial tissue volume

**Fig. 1:**
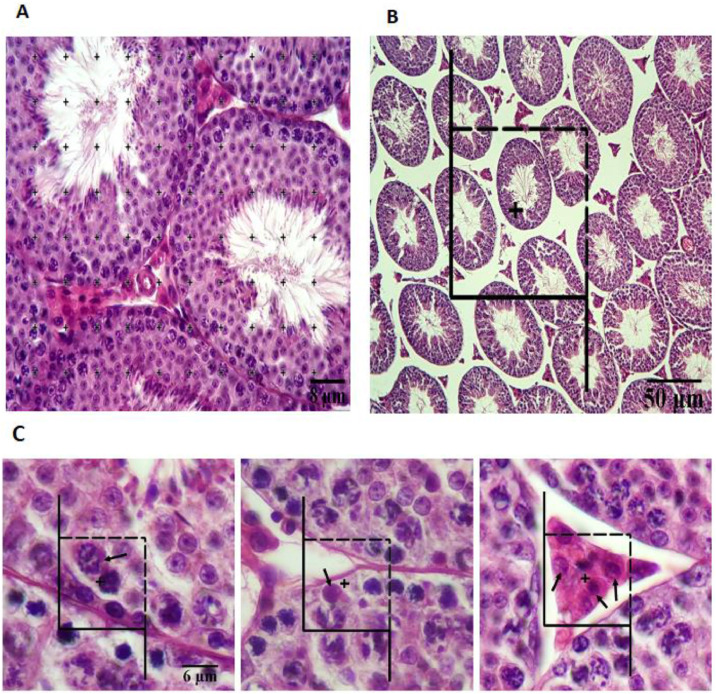
(A) An estimation of the volume density of the structures has been made using a point-counting technique. (B) An unbiased counting frame has been applied to the images to estimate the length of the seminiferous tubules. Six tubule profiles are counted here, which are inside the counting frame completely or partially, but only touching the top and right lines of the counting frame. The tubule profiles that touch the bottom and left lines, as well as their extensions, are not considered. (C) By using an optical dissector method, an unbiased counting frame is superimposed on the images to estimate the numerical density of different cells.

To calculate the total volume of the seminiferous tubules and interstitial tissue, five-micrometer sections were analyzed. To determine the volume density, represented as “Vv (structure/testis)” of either a tubule or interstitial tissue, a technique called point counting, as shown in Fig. 1A, was utilized at 160 × magnification using the formula: $structure/testis\;=\;\frac{\Sigma \;P(structure)}{\Sigma \;P(testis)}$. Here, P(structure) indicates the count of grid points covering the tubules or interstitial tissue, and P(testis) indicates the points covering the entire testis.^25^ Vv (structure/testis), or volume density, is the proportion of the testis volume occupied by a specific structure (e.g., tubules). From these counts and knowing the final volume of the testis, the absolute volume of the structure can be calculated using: V(structure)=Vv(structure/testis)×V(final); V(structure) is the absolute three-dimensional volume of a structure (e.g., total tubule volume) within the testis.

### Estimation of seminiferous tubular length

To determine the length density of the seminiferous tubules, a random counting frame was used on the monitor at a magnification of 180 × . The length density (Lv) of the tubules was calculated using the formula: $Lv\;=\;\frac{2\;\times \Sigma Q}{(a/f)\;\times \Sigma f}$. Here, “ΣQ” represents the total number of tubule profiles counted in each mouse testis, “a/f” denotes the area of the counting frame (422 × 422 µm), and “Σf” indicates the total number of frames counted for each animal. Lv, or length density, is the total length of tubules per unit volume of testicular tissue. To find the total length of the tubules (L), the volume of the tubules was multiplied by their length density (Lv). Fig. 1B illustrates the use of a random counting frame on the monitor at 180 × magnification to measure the length of the seminiferous tubules.^26^^,^^27^ L is the total summed length of all seminiferous tubules in the testis.

### Estimation of cell numbers in testis sections

Slices of tissue were prepared with a thickness of 20 micrometers, and various types of cells, such as Leydig cells, Sertoli cells, spermatogonia, spermatocytes, and round spermatids, were examined using an oil immersion lens with a high numerical aperture (NA = 1.4). To ensure accurate cell counting, a stereology software program was employed to overlay a counting frame on the images of testis sections displayed on a monitor. Cell numbers were estimated using a method known as the “optical dissector”, which helps avoid “edge effects” and biased counting (Fig. 1C). This method ensured that all cell nuclei within the frame had an equal chance of being counted. The optical section was adjusted downward along the z-axis. The top 5 ^µm^ of each section were excluded as a guard zone to prevent bias.

The formula used to calculate the numerical density of cells per testicle (Nv) is given as Nv(cells/testicle)=ΣQ/(ΣA×h), where “ΣQ” indicates the total nuclei counted within the dissector height, “ΣA” represents the area covered by the unbiased counting frame in each microscopic field (168 µm^2), and “h” denotes the height of the dissector, set at 5 micrometers. Nv, or numerical density, is the number of cells per unit volume of a reference space (e.g., testis or epithelium). By multiplying the numerical density (Nv) by the volume of the epithelium, the total number of nuclei was calculated.^25^

### Procedures for RNA isolation, cDNA preparation, and quantitative real-time RT-PCR analysis

Total RNA was extracted from testicular tissue using RNX Plus (Cinnagen, Iran), following the manufacturer's instructions. The QuantiTect Reverse Transcription Kit (Qiagen, Germany) was employed for synthesizing the first-strand cDNA as per the guidelines provided by the manufacturer. Quantitative real-time RT-PCR was conducted using an ABI Prism 7500 Sequence Detection System (Applied Biosystems, Foster City, CA, United States). The PCR amplification was carried out in a 25 µL reaction volume that included 1 µL of cDNA template, 1 µL of primer (10 pmoL/μL), and 12.5 µL of RealQ Plus 2 × Master Mix Green Low ROX (Ampliqon, Odense, Denmark). Glyceraldehyde 3-phosphate dehydrogenase (GAPDH) was used to normalize the target gene dosage levels. The study aimed to assess the expression levels of the antioxidant enzymes *Glutathione Peroxidase 1* (*GPx1*), *Superoxide dismutase-1* (*Sod1*), and *Catalase* (*Cat*), as well as the levels of *Bcl2l1* (an apoptotic inhibitor) and *Bax* (an apoptotic activator) in testicular samples. Sample analysis was performed using the 2^-ΔΔCt^ method. Table 1 lists the primers that were used for RT-PCR.

**Table 1:** A description of the primers used for quantitative real-time RT-PCR.

Genes	Primer Sequences		Product Size
*GPx1*	F (5′–3′)	CAGGAGAATGGCAAGAATGAAGAG	136 bp
	R (5′–3′)	GGAAGGTAAAGAGCGGGTGA	
*Sod1*	F (5′–3′)	GGGTTCCACGTCCATCAGTAT	121 bp
	R (5′–3′)	GGTCTCCAACATGCCTCTCTT	
*Cat*	F (5′–3′)	CTCAGGTGCGGACATTCTACA	206 bp
	R (5′–3′)	AATTGCGTTCTTAGGCTTCTCAG	
*Bcl2l1*	F (5′–3′)	GCAGGTATTGGTGAGTCGGA	130 bp
	R (5′–3′)	CTCGGCTGCTGCATTGTTC	
*Bax*	F (5′–3′)	TGGAGATGAACTGGACAGCAAT	155 bp
	R (5′–3′)	TAGCAAAGTAGAAGAGGGCAACC	
*Caspase3*	F (5′–3′)	TGACTGGAAAGCCGAAACTC	122 bp
	R (5′–3′)	AGCCTCCACCGGTATCTTCT	
*Gapdh*	F (5′–3′)	TGTTTCCTCGTCCCGTAGA	120 bp
	R (5′–3′)	ATCTCCACTTTGCCACTGC	

### Statistical analysis

The data are reported as mean and standard deviation. The mean and standard deviation are reported for the data. The one-way ANOVA and Tukey test were used to make statistical comparisons between the means of the groups. It was considered to be significant if the “p-value” was less than 0.05.

## Results

### The total volume of the seminiferous tubules and interstitial tissue

**Fig. 2:**
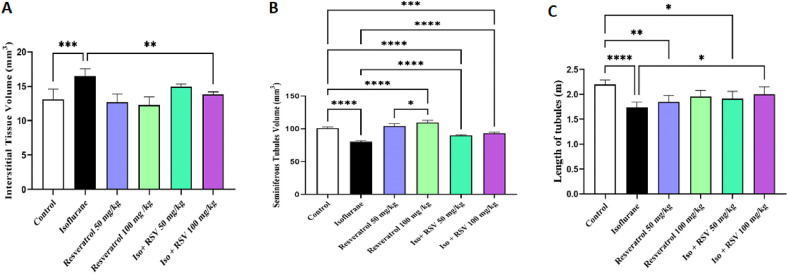
**Resveratrol mitigates the adverse effects of isoflurane on testicular morphology.** Stereological evaluation of (A) interstitial tissue volume, (B) seminiferous tubule volume, and (C) seminiferous tubule length, demonstrating that resveratrol treatment preserves testicular architecture against isoflurane challenge. Data are presented as mean ± SEM. * *p* < 0.05, ** *p* < 0.01, *** *p* < 0.001, and **** *p* < 0.0001.

A significant reduction in the seminiferous tubule volume was observed in isoflurane-treated mice compared to controls (*p* < 0.0001). Co-treatment with resveratrol at 50 mg/kg and 100 mg/kg completely prevented this atrophy, with tubule volumes being significantly higher than in the isoflurane group (*p* < 0.0001 for both doses) (Fig. 2).

Isoflurane treatment resulted in a significant expansion of the interstitial compartment compared to the control group (*p* < 0.001). This effect was significantly attenuated by resveratrol with the 100 mg/kg dose (*p* < 0.01), reducing the interstitial volume relative to the isoflurane-only group (Fig. 2).

### Seminiferous tubule length

Isoflurane treatment induced a significant reduction in seminiferous tubular length compared to the control group (*p* < 0.0001; Fig. 2). Co-treatment with resveratrol demonstrated a dose-dependent amelioration of this effect. While the low dose (50 mg/kg) showed non-significant improvement, the high (100 mg/kg) doses showed the most pronounced protective effect compared to the isoflurane-only group (*p* < 0.05) (Fig. 2).

### Quantification of various cell types in the testis

**Fig. 3:**
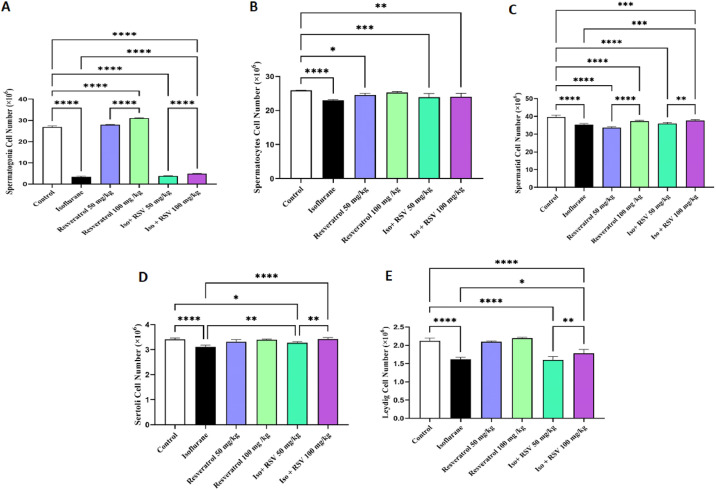
**Resveratrol prevents isoflurane-induced loss of testicular cells.** Stereological cell counts showing the population of (A) spermatogonia, (B) spermatocytes, (C) spermatids, (D) Sertoli cells, and (E) Leydig cells. Co-treatment with resveratrol significantly maintained cell numbers compared to the isoflurane-only group. Data are presented as mean ± SEM. * *p* < 0.05, ** *p* < 0.01, *** *p* < 0.001, and **** *p* < 0.0001.

In the isoflurane-exposed group, a significant reduction in the numbers of spermatogonia, spermatocytes, round spermatids, Leydig cells, and Sertoli cells was observed compared with the control group (*p* < 0.0001). The adverse effects on cell counts were ameliorated when high doses of resveratrol were combined with isoflurane, leading to an increase in spermatogonia, spermatocytes, round spermatids, Leydig cells, and Sertoli cells (*p* < 0.0001, *p* < 0.001, *p* < 0.0001, and *p* < 0.05, respectively). No significant differences were found in the number of spermatocytes between the animals treated with both high and low doses of resveratrol and isoflurane and those treated with isoflurane alone (*p* > 0.05). Notably, a significant elevation in the number of Sertoli cells was recorded in the group receiving a low dose of resveratrol and isoflurane compared to the isoflurane-only group (*p* < 0.01) (Fig. 3).

### Expression of apoptotic and antioxidant genes

Gene expression analysis revealed that isoflurane exposure significantly disrupted the balance of apoptosis-related factors, marked by a decrease in the anti-apoptotic gene *Bcl2l1* and an increase in the pro-apoptotic genes *Bax* and Caspase-3 compared to the control (*p* < 0.0001). Co-treatment with a high dose of resveratrol (100 mg/kg) significantly counteracted these effects, reducing *Bax* and Caspase-3 and increasing *Bcl2l1* levels relative to the isoflurane-only group (*p* < 0.0001, *p* < 0.01, and *p* < 0.01, respectively)

**Fig. 4:**
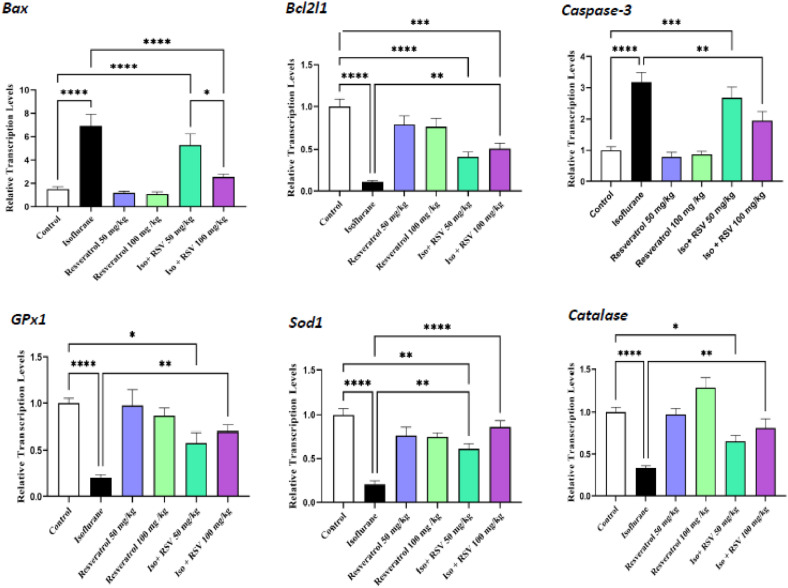
**Resveratrol alters the expression of key genes involved in apoptosis and oxidative stress in isoflurane-exposed testes.** Relative mRNA expression of (A) pro-apoptotic and anti-apoptotic genes and (B) antioxidant enzymes. Resveratrol treatment reversed the isoflurane-induced gene expression profile. Data are presented as mean ± SEM. * *p* < 0.05, ** *p* < 0.01, *** *p* < 0.001, and **** *p* < 0.0001.

The transcription levels of *GPx1, Sod1*, and catalase significantly reduced in the Isoflurane group compared to the control group (*p* < 0.0001), while combination therapy with high doses of resveratrol increased their expression levels compared to the Isoflurane group (*p* < 0.01, *p* < 0.0001, and *p* < 0.01). It was observed that if 50 mg/kg resveratrol was administered in combination with isoflurane, it was only able to significantly increase the expression level of the *Sod1* gene over the isoflurane group (*p* < 0.01) (Fig. 4).

## Discussion

It is widely acknowledged that oxidative stress is a significant contributing factor to male infertility.^28^ The accumulation of reactive oxygen species ROS in testicular tissue, which can overwhelm endogenous antioxidant defenses, is a key driver of apoptosis and cellular damage, ultimately compromising reproductive function.^29^ Natural compounds, such as resveratrol, offer a promising therapeutic approach by targeting oxidative stress, apoptosis, and hormonal balance.^30^ This study was conducted to investigate the therapeutic role of resveratrol, a compound with known antioxidant and anti-inflammatory properties, against isoflurane-induced testicular toxicity.

These findings demonstrate that isoflurane exposure induces significant testicular injury, characterized by an enhancement of interstitial tissue and a reduction in the volume and length of the seminiferous tubules. These morphological changes are consistent with the authors’ previous reports of testicular weight reduction and germinal epithelial cell atrophy following exposure to isoflurane.^31^ The authors propose that these structural deficits are a direct consequence of disrupted spermatogenesis, evidenced by a reduction in germ cell populations and increased apoptosis.^32^^,^^33^

A key mechanism underlying this damage appears to be the induction of oxidative stress and a shift in the balance towards apoptosis. In the isoflurane group, the authors observed a significant decrease in the gene expression of the anti-apoptotic protein *Bcl2l1*, alongside an increase in the pro-apoptotic markers *Bax* and Caspase-3. Concurrently, isoflurane likely disrupts the redox balance, creating a pro-oxidant environment. Resveratrol treatment effectively ameliorated these adverse effects.

The isoflurane-induced decrease in various testicular cell populations was reversed, particularly with high-dose resveratrol, a finding consistent with studies showing resveratrol's ability to improve spermatogenic cell counts.^34^, ^35^, ^36^ According to these results, the protective mechanism of resveratrol is multifaceted. Firstly, it functions as a potent antioxidant, upregulating the gene expression of key antioxidant enzymes such as *GPx1, Sod1*, and catalase to restore ROS equilibrium. Secondly, it exerts strong anti-apoptotic effects, counteracting the isoflurane-induced expression of *Bax* and Caspase-3. This aligns with established literature demonstrating that resveratrol can protect against testicular damage by reducing mitochondrial-mediated apoptosis and inflammation.^21^^,^^37^

These cellular and molecular improvements translate to enhanced reproductive function. The authors’ previous work has shown that resveratrol mitigates the negative effects of isoflurane on sperm parameters, including motility, viability, and DNA integrity.^31^ By reducing oxidative stress and apoptosis, resveratrol preserves the integrity of the seminiferous epithelium and supports normal spermatogenesis. This protective role is further supported by evidence that resveratrol can increase testosterone production,^35^ a hormone critical for maintaining the structural and functional integrity of the seminiferous tubules.

This study has several limitations. Although the authors established a strong association between resveratrol and the amelioration of testicular damage, the precise underlying molecular pathways were not fully elucidated. For instance, the specific signaling cascades responsible for its anti-apoptotic and antioxidant effects remain to be defined. Furthermore, the present study utilized a preventive model, where resveratrol was administered concurrently with isoflurane; thus, its potential as a therapeutic intervention after anesthetic exposure remains unknown. Future studies should incorporate different animal models, more detailed mechanistic investigations, and varied treatment schedules to confirm and expand upon these findings.

## Conclusions

In conclusion, isoflurane induces testicular damage through pathways involving oxidative stress and apoptosis, leading to morphological degeneration and impaired spermatogenesis. Resveratrol emerges as a highly effective cytoprotective agent in this model, mitigating this damage through its complementary antioxidant and anti-apoptotic properties. This study provides compelling evidence for the potential of resveratrol as a therapeutic intervention to safeguard male reproductive health against anesthetic-induced injury.

## Ethics approval and consent to participate

All experimental procedures involving animals were approved by the Animal Ethics Committee of Shiraz University of Medical Sciences (Approval ID: IR.SUMS.AEC.1402.060) and conducted in compliance with the ARRIVE guidelines (Animal Research: Reporting of In Vivo Experiments) to ensure rigorous reporting, transparency, and reproducibility.

## Consent for publication

Not applicable

## Authors' contributions

The conceptualization of the project was carried out by Zahra Mohammadi and Sanaz Alaee, while formal analysis was conducted by Majid Kamali-Dolatabadi, Somayyeh Karami-Mohajeri and Zahra Khodabandeh, Saeed Shokri, Sulagna Dutta, and Pallav Sengupta. Funding acquisition was managed by Sanaz Alaee. The writing of the original draft was done by Zahra Mohammadi and Sanaz Alaee, followed by review and editing by Pallav Sengupta and Hesam Kamyab.

## Data availability statement

All data generated or analyzed during this study are included in this published article.

## Declaration of competing interest

The authors declare no conflicts of interest.
